# The prevalence of obesity-related hypertension among middle-aged and older adults in China

**DOI:** 10.3389/fpubh.2022.865870

**Published:** 2022-11-24

**Authors:** Yang Zhang, Wen-Qiang Zhang, Wei-Wei Tang, Wen-Yong Zhang, Jian-Xiong Liu, Rong-Hua Xu, Tzung-Dau Wang, Xiao-Bo Huang

**Affiliations:** ^1^Department of Cardiology, Chengdu Second People's Hospital, Chengdu, China; ^2^Department of Epidemiology and Health Statistics, West China School of Public Health and West China Fourth Hospital, Sichuan University, Chengdu, China; ^3^School of Health Policy and Management, Nanjing Medical University, Nanjing, China; ^4^Institute of Healthy Jiangsu Development, Nanjing Medical University, Nanjing, China; ^5^Stroke Center, Chengdu Second People's Hospital, Chengdu, China; ^6^Division of Cardiology, Department of Internal Medicine, National Taiwan University Hospital, Taipei City, Taiwan

**Keywords:** prevalence, obesity-related hypertension, hypertension, obesity, China, risk factors

## Abstract

**Objective:**

The aim of our study was to assess the prevalence and geographic variation of obesity-related hypertension in China among adults aged 45 years or older.

**Methods:**

Data were derived from the China Health and Retirement Longitudinal Study (CHARLS) conducted in 2015. Stratified sample households covered 150 counties/districts and 450 villages/urban communities from 28 provinces by using household questionnaires, clinical measurements, and blood-based bioassays. A multivariable non-conditional logistic regression model was used to analyze the risk factors correlated with obesity-related hypertension.

**Results:**

The prevalence of obesity-related hypertension was 22.7%, ~120 million people, among adults aged 45 years or older in China. For people in the age ranges of 45–54, 55–64, 65–74, and ≥75 years, the prevalence of obesity-related hypertension was 16.7, 24.3, 27, and 26.7%, respectively, and the prevalence of obesity-related hypertension among hypertensive participants was 66.0, 60.9, 54.2, and 47.3%, respectively. Compared with non-obesity-related hypertension, the obesity-related hypertensive patients had a higher prevalence of diabetes mellitus, dyslipidemia, and hyperuricemia (all *P* < 0.0001). The prevalence of obesity-related hypertension showed a decreasing gradient from north to south and from east to west. Multivariate logistic regression analysis showed that female gender, living in urban areas, diabetes mellitus, dyslipidemia, and hyperuricemia were positively correlated with obesity-related hypertension.

**Conclusion:**

The prevalence of obesity-related hypertension among adults aged 45 years or older was high in China. Among hypertensive participants, older age was negatively correlated with obesity-related hypertension. Obesity-related hypertensive participants are more prone to aggregation of risk factors of atherosclerotic cardiovascular disease.

## Introduction

Hypertension and obesity are independent risk factors for cardiovascular diseases and all-cause mortality (1–4), and they often co-existed (5, 6). In the past few decades, China has seen rapid economic development and urbanization, which have led to lifestyle changes such as increased unhealthy nutrition and reduced physical activity, which in turn have led to an increase in hypertension and obesity prevalence (1, 7, 8). The prevalence of hypertension increased from 18% in 2002 (9) to 29.6% in 2010 (10) among Chinese adults. The prevalence of general obesity (body mass index, BMI ≥ 28 kg/m^2^) increased from 4.0 in 2002 to 10.7% in 2009 (11). In the 2011 China Health and Nutrition Survey, the prevalence of general obesity (BMI ≥ 28 kg/m^2^) increased to 11.3% (12). From 1993 to 2015, mean waist circumference (WC) increased from 76.0 to 83.4 cm and the prevalence increased from 20.2 to 46.9% for abdominal obesity (WC ≥90 cm for men and ≥80 cm for women) (13). In the China Health and Nutrition Survey (CHNS) conducted from 1993 to 2009, the trend in the prevalence of abdominal (central) obesity among participants with normal BMI (BMI < 25 kg/m^2^) increased from 11.9 to 21.1% (14).

Previous studies revealed that the prevalence of hypertension was more than two times higher in general obesity than in non-obese individuals (15), and abdominal obesity patients had a 2–3-fold higher risk of developing hypertension than non-obese individuals (16). Hypertensive patients with obesity are prone to requiring more antihypertensive medications and have a higher risk of developing treatment-resistant hypertension (6). Furthermore, they are more likely to be exposed to metabolic and cardiovascular risk factors (17). From 2009 to 2012, the European Society of Hypertension Working Group on Obesity published a series of articles about the pathophysiology, cardiovascular risk, target organ damage, and treatment of obesity-related hypertension (6, 18–20). In 2013, the Obesity Society and the American Society of Hypertension agreed to jointly sponsor a position paper: Obesity-related hypertension: pathogenesis, cardiovascular risk, and treatment (21). In 2016, China published the “Chinese expert consensus on obesity-related hypertension management” to provide definitions and guidelines regarding the treatment of obesity-related hypertension (22). More and more attention has been paid to the diagnosis and treatment of obesity-related hypertension. However, there has been no national epidemiological investigation into it. Our study aimed at reporting the prevalence and regional distribution of obesity-related hypertension and its characteristics among adults aged 45 years or older in China.

## Methods

### Participants

China Health and Retirement Longitudinal Study (CHARLS) is a nationally representative survey of adults aged 45 years or older, including household questionnaires, clinical measurements, and blood-based bioassays. CHARLS samples covered 150 counties/districts and 450 villages/urban communities based on probability proportional to size random sampling procedures. In the study, 13,013 respondents provided venous blood samples, of which the shipping temperature was strictly controlled. Blood sample analysis was carried out in two stages. Blood samples were analyzed by whole blood cell count in the local county health centers immediately. Then the samples were transported to the headquarters, where they were analyzed for hsCRP, HbA1c, blood lipids, blood glucose, blood urea nitrogen, creatinine, and uric acid. Before the blood pressure test, respondents were relaxed and kept sitting. The blood pressure was measured using an Omron hem-7,200 every 5 min, and the blood pressure was measured three times. We selected 11,364 participants who donated fasting blood samples, assayed on a whole range of indicators, and were interviewed in the 2015 follow-up wave of CHARLS. We excluded individuals if they did not complete clinical measurements and blood-based bioassays; or they had missing values for sex, birth date, residence, marital status, weight, height, waist circumference, and blood pressure; or they had unreliable values for weight, height, waist circumference; or they were < 45 years old. A total of 10,108 participants were included in the final analysis. All bioassays were performed at KingMed Laboratory. The biomarker sample collection study protocol was approved by the Ethical Review Committee (Institutional Review Board) of Peking University (IRB 00001052-11014). Written informed consent was obtained from all study participants.

### Definition of obesity-related hypertension

We defined obesity-related hypertension as (1) body mass index (BMI) ≥ 28 kg/m^2^ and/or waist circumstance (WC) ≥ 90 cm (male) or 85 cm (female) and (2) systolic blood pressure (SBP) ≥ 140 mmHg and/or diastolic blood pressure (DBP) ≥ 90 mmHg or a self-reported diagnosis of hypertension, according to Chinese expert consensus on the obesity-related hypertension management (22).

### Definition of independent variable

Married or cohabitated was defined as any one of the following: (1) married with spouse present, (2) married but not living with spouse temporarily for reasons such as work, and (3) cohabitated. Singles included those who were separated, divorced, widowed, or never married. Urban areas included (1) main city zone, (2) combination zone between urban and rural areas, (3) the town center, (4) ZhenXiang area, and (5) special area. Rural areas included (1) township central and (2) village. The Qinling Mountains and Huaihe River line are considered the natural dividing line between north and south China (23), in this study. Northern provinces included Beijing, Gansu, Hebei, Henan, Heilongjiang, Jilin, Liaoning, Inner Mongolia autonomous region, Qinghai, Shandong, Shaanxi, Shanxi, Tianjin, and Xinjiang Uygur Autonomous Region. Southern provinces included Anhui, Fujian, Guangdong, Guangxi Zhuang Autonomous Region, Guizhou, Hubei, Hunan, Jiangsu, Jiangxi, Shanghai, Sichuan, Yunnan, Zhejiang, and Chongqing. In combination with the seventh five-year plan adopted at the sixth session of the National People's Congress and the western development policy formulated by the state, China is divided into three belts: the central, the east, and the west. In this study, Central provinces included Anhui, Henan, Heilongjiang, Hubei, Hunan, Jilin, Jiangxi, and Shanxi. Eastern provinces included Beijing, Fujian, Guangdong, Hebei, Jiangsu, Liaoning, Shandong, Shanghai, Tianjin, and Zhejiang. Western provinces included Gansu, Guangxi Zhuang Autonomous Region, Guizhou, Inner Mongolia autonomous region, Qinghai, Shaanxi, Sichuan, Xinjiang Uygur Autonomous Region, Yunnan, and Chongqing.

Smoking was defined as any one of the following: (1) having ever chewed tobacco, (2) smoked a pipe, (3) smoked self-rolled cigarettes, or (4) smoked cigarettes/cigars. Drinking was defined as any alcoholic beverage consumed in the past year. Diabetes mellitus was defined as any one of the following: (1) a self-reported diagnosis of diabetes mellitus, (2) fasting plasma glucose ≥ 126 mg/dl (7.0 mmol/L), and (3) glycated hemoglobin (HbA1c) ≥ 6.5% (24). Dyslipidemia was defined as any one of the following: (1) a self-reported diagnosis of dyslipidemia, (2) total cholesterol (TC) ≥ 240 mg/dl (6.2 mmol/L), (3) low-density lipoprotein cholesterol (LDL-C) ≥ 160 mg/dl (4.1 mmol/L), (4) high-density lipoprotein cholesterol (HDL-C) < 40 mg/dl (1.0 mmol/L), or (5) triglyceride (TG) ≥ 200 mg/dl (2.3 mmol/L) (25). Hyperuricemia was defined as male serum uric acid (SUA) > 7 mg/dl (420 μmol/L) and female SUA > 6 mg/dL (360 μmol/L) (26).

### Statistical analysis

Quantitative data with a normal distribution were presented as a mean with the standard deviation (SD), and the Student's *t-*test was used to detect the difference across the groups. Quantitative data with the skewed distribution were presented as medians with the interquartile range, and the Wilcoxon rank sum test was used to detect the difference across the groups. Qualitative data were presented as absolute numbers with the percentage (%), Pearson's chi-square test was used to assess the statistical significance of differences, and the Cochran-Armitage trend test was performed to support the trend hypothesis. We estimated the prevalence of hypertension and obesity-related hypertension for both men and women in four age groups (45–54, 55–64, 65–74, and ≥75 years) in rural and urban settings. Furthermore, we created a dump variable for missing values. A multivariable non-conditional logistic regression model was used to estimate the odds ratios and 95% confidence interval (95% CI) to explore the associated risk factors of obesity-related hypertension. We performed all the statistical analyses using SAS version 9.4 (SAS Institute, Cary, NC, USA). A *P* < 0.05 was considered significant.

## Results

### The prevalence of obesity, hypertension, and obesity-related hypertension in different populations

#### All participants

**Figure 1:**
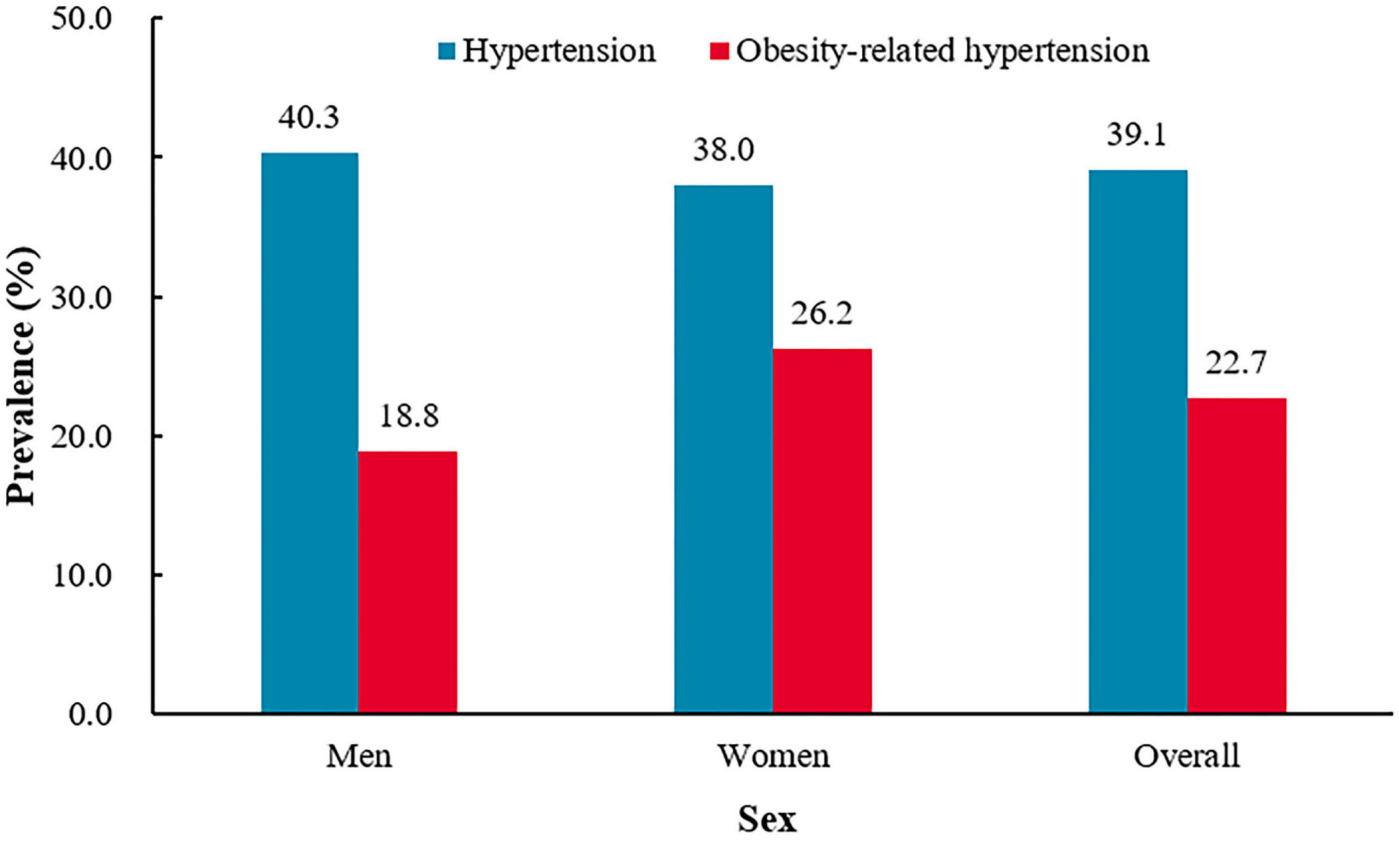
The prevalence of hypertension and obesity-related hypertension among China adults aged 45 years or older.^†^*P* = 0.0179 for sex difference in the prevalence of hypertension.^‡^*P* = 0.0001 for sex difference in the prevalence of obesity-related hypertension.

**Figure 2:**
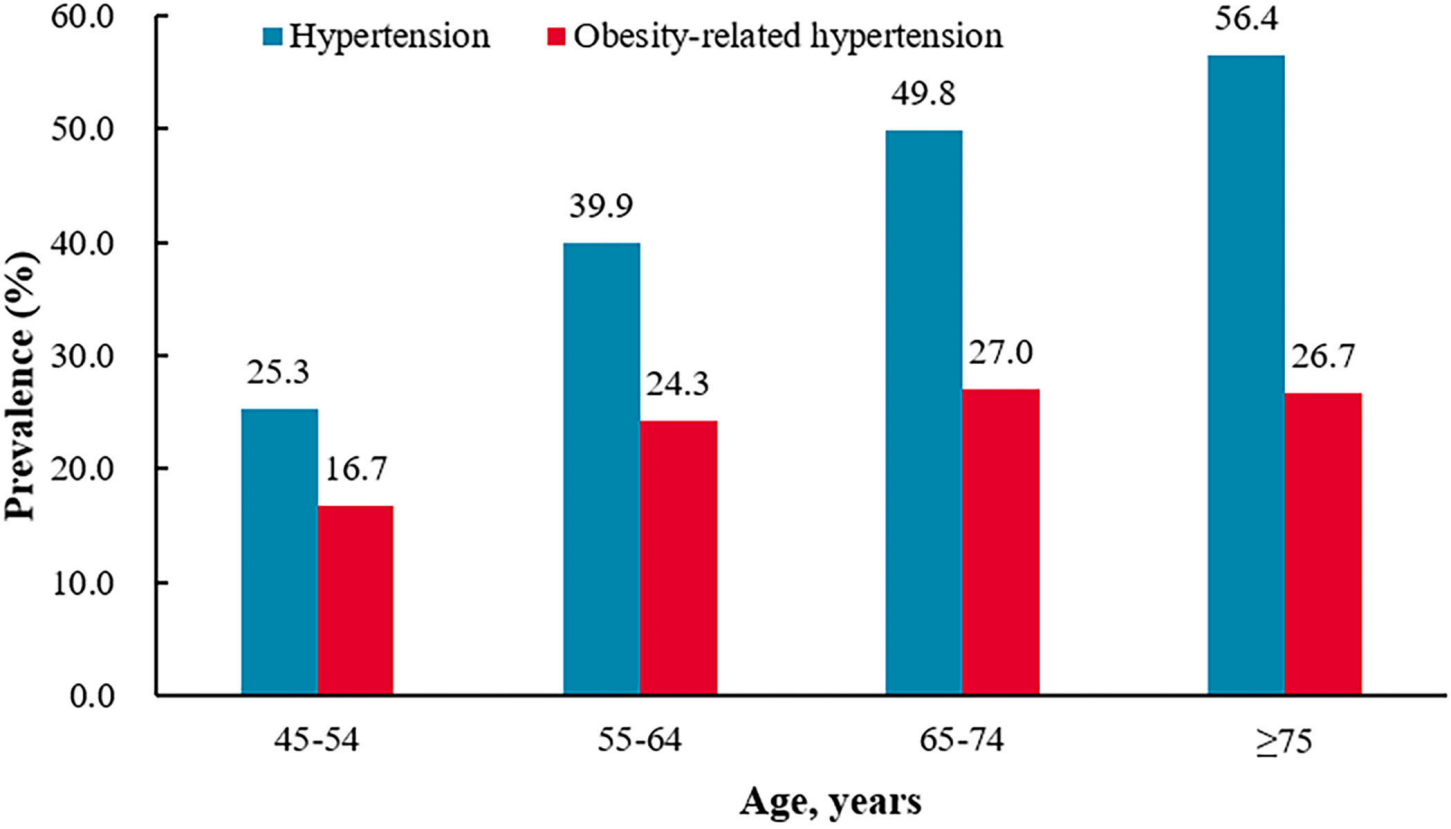
The prevalence of hypertension and obesity-related hypertension different age groups.^†^*P*-trend<0.0001 for the trend in the prevalence of hypertension by age groups.^‡^*P*-trend<0.0001 for the trend in the prevalence of obesity-related hypertension by age groups. *P = 0.8806 for the difference in the prevalence of obesity-related hypertension between 65–74 age group and >75 age group.

A total of 10,108 participants aged 45 years or older were included in the final analysis, among whom 4,705 were men (46.5%) and 5,403 were women (53.5%). From the western provinces to the central provinces to the eastern provinces, the participants were 3,096 (30.6%), 3,627 (35.9%), 3,385 (33.5%), respectively. From the northern provinces to the southern provinces, the participants were 4,841 (47.9%) vs. 5,267 (52.1%). The prevalence of hypertension, obesity, and obesity-related hypertension among all participants was 39.1% (3,951/10,108), 47.3% (4,783/10,108), and 22.7% (2,299/10,108), respectively. The prevalence of obesity-related hypertension among obese and hypertensive participants was 48.1% (2,299/4,783) and 58.2% (2,299/3,951), respectively. The prevalence of hypertension increased with age (*P* < 0.001). The prevalence of obesity-related hypertension increased with age (*P* < 0.001), but it peaked at ages 65–74 years. The prevalence of obesity-related hypertension among hypertensive participants decreased with age (Figures 1, 2, Tables 1–3).

**Table 1:** Basic characteristics of the study participants.

**Characteristics**	**Total** **(*N* = 10,108)**	**Normal population (*N* = 3,673)**	**Obesity without hypertension (*N* = 2,484)**	**Non-obesity-related hypertension (*N* = 1,652)**	**Obesity-related hypertension (*N* = 2,299)**	***P*** **-values**
Sex						<0.001
Men	4,705 (46.5)	1,997 (53.8)	831 (33.5)	1,014 (61.4)	883 (38.4)	
Women	5,403 (53.5)	1,696 (46.2)	1,653 (66.5)	638 (38.6)	1,416 (61.6)	
Age, years						<0.001
45–54	3,230 (32.0)	1,376 (37.5)	1,037 (41.7)	279 (16.9)	538 (23.4)	
55–64	3,515 (34.8)	1,255 (34.2)	858 (34.5)	547 (33.1)	855 (37.2)	
65–74	2,503 (24.8)	781 (21.3)	475 (19.1)	571 (34.6)	676 (29.4)	
≥75	860 (8.5)	261 (7.1)	114 (4.6)	255 (15.4)	230 (10.0)	
Marital status						<0.001
Married or cohabitated	8,792 (87.0)	3,265 (88.9)	2,226 (89.6)	1,354 (82.0)	1,947 (84.7)	
Single	1,316 (13.0)	408 (11.1)	258 (10.4)	298 (18.0)	352 (15.3)	
Residence						<0.001
Urban	2,447 (24.2)	765 (20.8)	725 (29.2)	308 (18.6)	649 (28.2)	
Rural	7,661 (75.8)	2,908 (79.2)	1,759 (70.8)	1,344 (81.4)	1,650 (71.8)	
Region 1						<0.001
Western	3,096 (30.6)	1,285 (35.0)	681 (27.4)	539 (32.6)	591 (25.7)	
Central	3,627 (35.9)	1,314 (35.8)	918 (37.0)	578 (35.0)	817 (35.5)	
Eastern	3,385 (33.5)	1,074 (29.2)	885 (35.6)	535 (32.4)	891 (38.8)	
Region 2						<0.001
Northern	4,841 (47.9)	1,500 (40.8)	1,315 (52.9)	694 (42.0)	1,332 (57.9)	
Southern	5,267 (52.1)	2,173 (59.2)	1,169 (47.1)	958 (58.0)	967 (42.1)	

Values are presented as number (%).

**Table 2:** The prevalence of obesity, hypertension, obesity-related of hypertension among men, women, urban and rural.

	**Overall**	**Men**	**Women**	***P*** **-Value**	**Urban**	**Rural**	***P*** **-Value**
HBP/all	39.1%(3,951/10,108)	40.3% (1,897/4,705)	38.0% (2,054/5,403)	0.0179	39.1% (957/2,447)	39.1% (2,994/7,661)	0.9802
Obesity/all	47.3% (4,783/10,108)	36.4% (1,714/4,705)	56.8% (3,069/5,403)	<0.0001	56.2% (1,374/2,447)	44.5% (3,409/7,661)	<0.0001
OB-HBP/all	22.7% (2,299/10,108)	18.8% (883/4,705)	26.2% (1,416/5,403)	<0.0001	26.5% (649/2,447)	21.5% (1,650/7,661)	<0.0001
OB-HBP/HBP	58.2% (2,299/3,951)	46.5% (883/1,897)	68.9% (1,416/2,054)	<0.0001	67.8% (649/957)	55.1% (1,650/2,994)	<0.0001
OB-HBP/OB	48.1% (2,299/4,783)	51.5% (883/1,714)	46.1% (1,416/3,069)	0.0004	47.2% (649/1,374)	48.4% (1,650/3,409)	0.4648

**Table 3:** The prevalence of hypertension and obesity-related of hypertension among different sex, age, residence, region.

**Characteristics**	**Hypertension**		**Obesity-related hypertension**	
	**Prevalence (%)**	***P***	**Prevalence (%)**	***P***
Total	3,951 (39.1)		2,299 (22.7)	
Sex		0.0179		< 0.0001
Men	1,897 (40.3)		883 (18.8)	
Women	2,054 (38.0)		1,416 (26.2)	
Age, years		< 0.0001		< 0.0001
45–54	817 (25.3)		538 (16.7)	
55–64	1,402 (39.9)		855 (24.3)	
65–74	1,247 (49.8)		676 (27.0)	
≥75	485 (56.4)		230 (26.7)	
Residence		0.9802		< 0.0001
Urban	957 (39.1)		649 (26.5)	
Rural	2,994 (39.1)		1,650 (21.5)	
Region 1		< 0.0001		< 0.0001
Western	1,130 (36.5)		591 (19.1)	
Central	1,395 (38.5)		817 (22.5)	
Eastern	1,426 (42.1)		891 (26.3)	
Region 2		< 0.0001		< 0.0001
Northern	2,026 (41.9)		1,332 (27.5)	
Southern	1,925 (36.5)		967 (18.4)	

#### Men vs. women

The prevalence of hypertension was higher in men (men 40.3% vs. women 38.0%, *P* = 0.0179). Women had a higher prevalence of obesity (men 36.4% vs. women 56.8%, *P* < 0.0001) and obesity-related hypertension (men 18.8% vs. women 26.2%, *P* < 0.0001). The prevalence of obesity-related hypertension was higher in women among hypertensive participants (men 46.5% vs. women 68.9%, *P* < 0.0001), but among the obese participants, the prevalence was higher in men (men 51.5% vs. women 46.1%, *P* = 0.0004) (Figure 1 and Table 2).

#### Urban areas vs. rural areas

**Figure 3:**
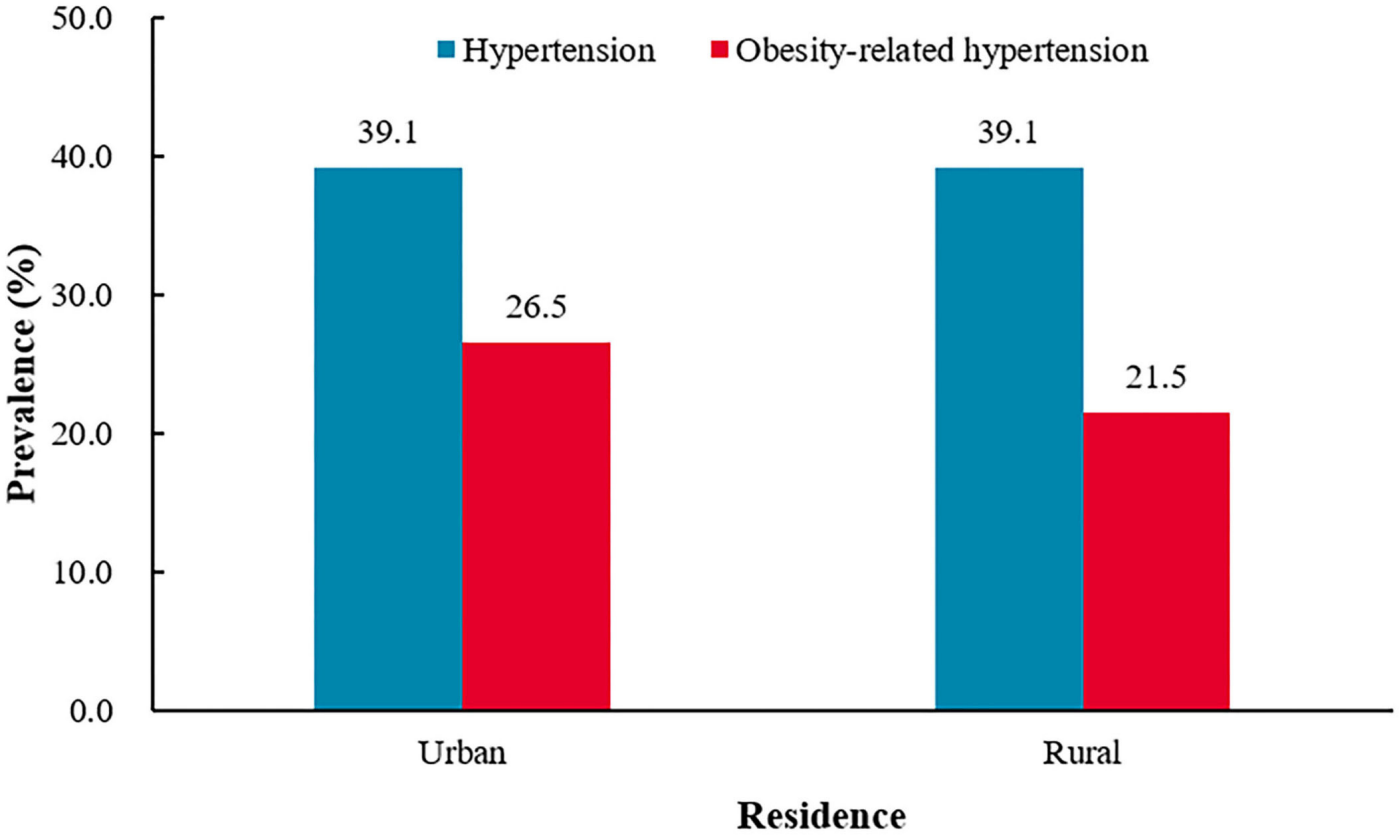
The prevalence of hypertension and obesity-related hypertension among urban and rural areas.^†^*P* = 0.9802 for the difference in the prevalence of hypertension between urban and rural.^‡^*P* < 0.0001 for the difference in the prevalence of obesity-related hypertension between urban and rural.

The prevalence of hypertension was similar between urban residents and rural residents (urban 39.1% vs. rural 39.1%, *P* = 0.9802). Compared with rural residents, urban residents had a higher prevalence of obesity (urban 56.2% vs. rural 44.5%, *P* < 0.0001) and obesity-related hypertension (urban 26.5% vs. rural 21.5%, *P* < 0.0001). The prevalence of obesity-related hypertension was higher in urban residents among hypertensive participants (urban 67.8% vs. rural 55.1%, *P* < 0.0001), but among obesity participants, the prevalence was similar between urban residents and rural residents (urban 47.2% vs. women 48.4%, *P* = 0.4648) (Figure 3 and Table 2).

#### Western provinces vs. central provinces vs. eastern provinces

**Figure 4:**
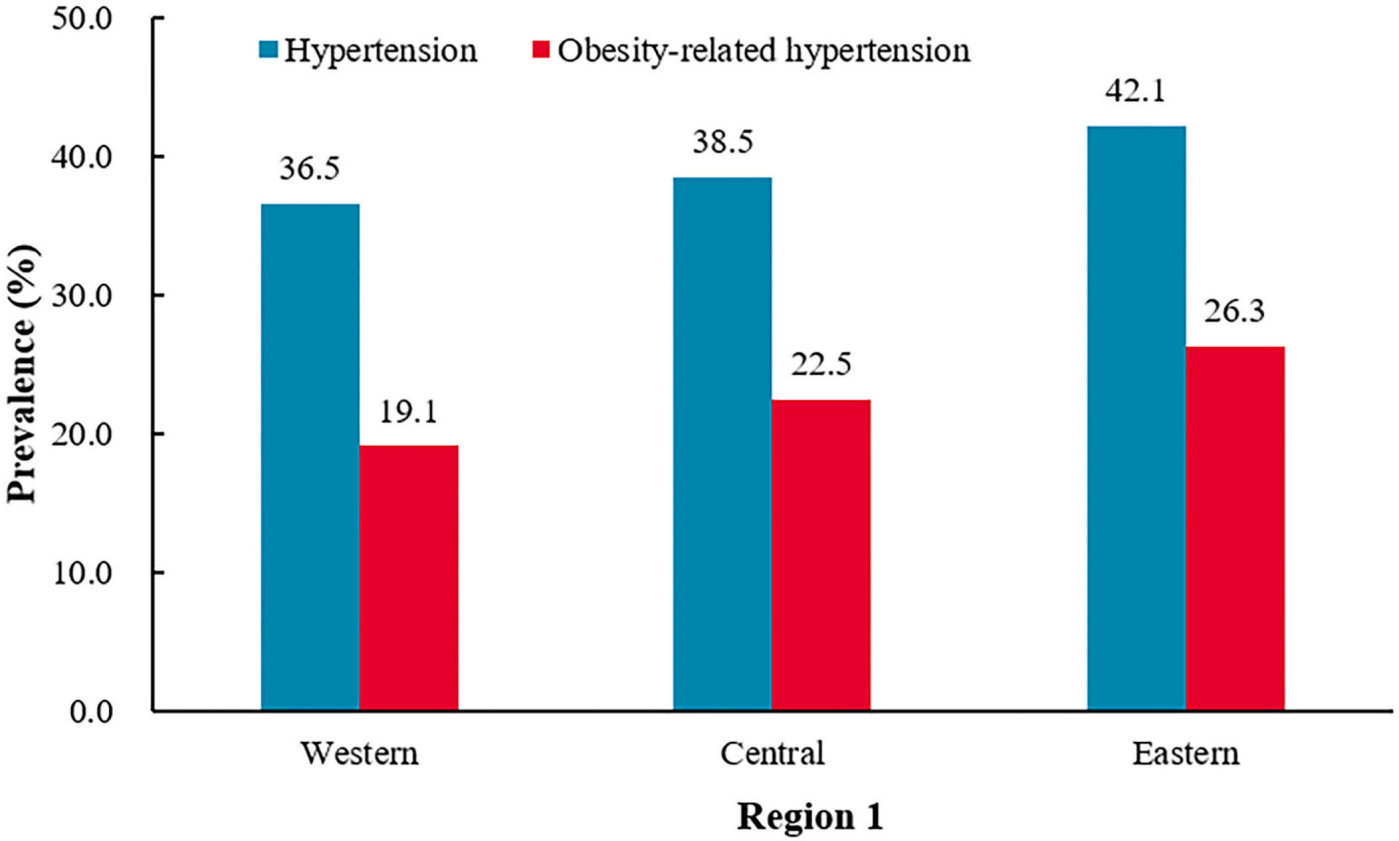
The prevalence of hypertension and obesity-related hypertension among Western, Central, and Eastern provinces.^†^*P* < 0.0001 for the difference in the prevalence of hypertension between Western, Central, and Eastern provinces.^‡^*P* < 0.0001 for the difference in the prevalence of obesity-related hypertension between Western, Central, and Eastern provinces.

From the western provinces to the central provinces to the eastern provinces, the prevalence of hypertension was 36.5, 38.5, and 42.1% (*P* < 0.0001), and the prevalence of obesity was 41.1, 47.8, and 52.5% (*P* < 0.0001), and the prevalence of obesity-related hypertension was 19.1, 22.5, and 26.3% (*P* < 0.0001), respectively. From western to eastern provinces, there was an increasing trend for the prevalence of hypertension, obesity, and obesity-related hypertension. From the western provinces to the central provinces to the eastern provinces, the prevalence of obesity-related hypertension among hypertensive participants was 52.3, 58.6, and 62.5% (*P* < 0.0001), and among obesity participants were 46.5, 47.1, and 50.2% (*P* = 0.0347), respectively (Figure 4 and Table 4).

**Table 4:** The prevalence of obesity, hypertension, obesity-related of hypertension among different regions.

	**Western**	**Central**	**Eastern**	***P*** -Value^*^	**Northern**	**Southern**	***P*** **-Value**
HBP/all	36.5% (1,130/3,096)	38.5% (1,395/3,627)	42.1% (1,426/3,385)	<0.0001	41.9% (2,026/4,841)	36.5% (1,925/5,267)	<0.0001
Obesity/all	41.1% (1,272/3,096)	47.8% (1,735/3,627)	52.5% (1,776/3,385)	<0.0001	54.7% (2,647/4,841)	40.6% (2,136/5,267)	<0.0001
OB-HBP/all	19.1% (591/3,096)	22.5% (817/3,627)	26.3% (891/3,385)	<0.0001	27.5% (1,332/4,841)	18.4% (967/5,267)	<0.0001
OB-HBP/HBP	52.3% (591/1,130)	58.6% (817/1,395)	62.5% (891/1,426)	<0.0001	65.7% (1,332/2,026)	50.2% (967/1,925)	<0.0001
OB-HBP/OB	46.5% (591/1,272)	47.1% (817/1,735)	50.2% (891/1,776)	0.0347	50.3% (1,332/2,647)	45.3% (967/2,136)	0.0005

* Cochran-Armitage trend test.

Northern provinces included Beijing, Gansu, Hebei, Henan, Heilongjiang, Jilin, Liaoning, Inner Mongolia autonomous region, Qinghai, Shandong, Shaanxi, Shanxi, Tianjin, Xinjiang Uygur Autonomous Region. Southern provinces included Anhui, Fujian, Guangdong, Guangxi Zhuang Autonomous Region, Guizhou, Hubei, Hunan, Jiangsu, Jiangxi, Shanghai, Sichuan, Yunnan, Zhejiang, Chongqing.

Western provinces included Gansu, Guangxi Zhuang Autonomous Region, Guizhou, Inner Mongolia autonomous region, Qinghai, Shaanxi, Sichuan, Xinjiang Uygur Autonomous Region, Yunnan, Chongqing. Central provinces included Anhui, Henan, Heilongjiang, Hubei, Hunan, Jilin, Jiangxi, Shanxi. Eastern provinces included Beijing, Fujian, Guangdong, Hebei, Jiangsu, Liaoning, Shandong, Shanghai, Tianjin, Zhejiang.

#### Northern provinces vs. southern provinces

**Figure 5:**
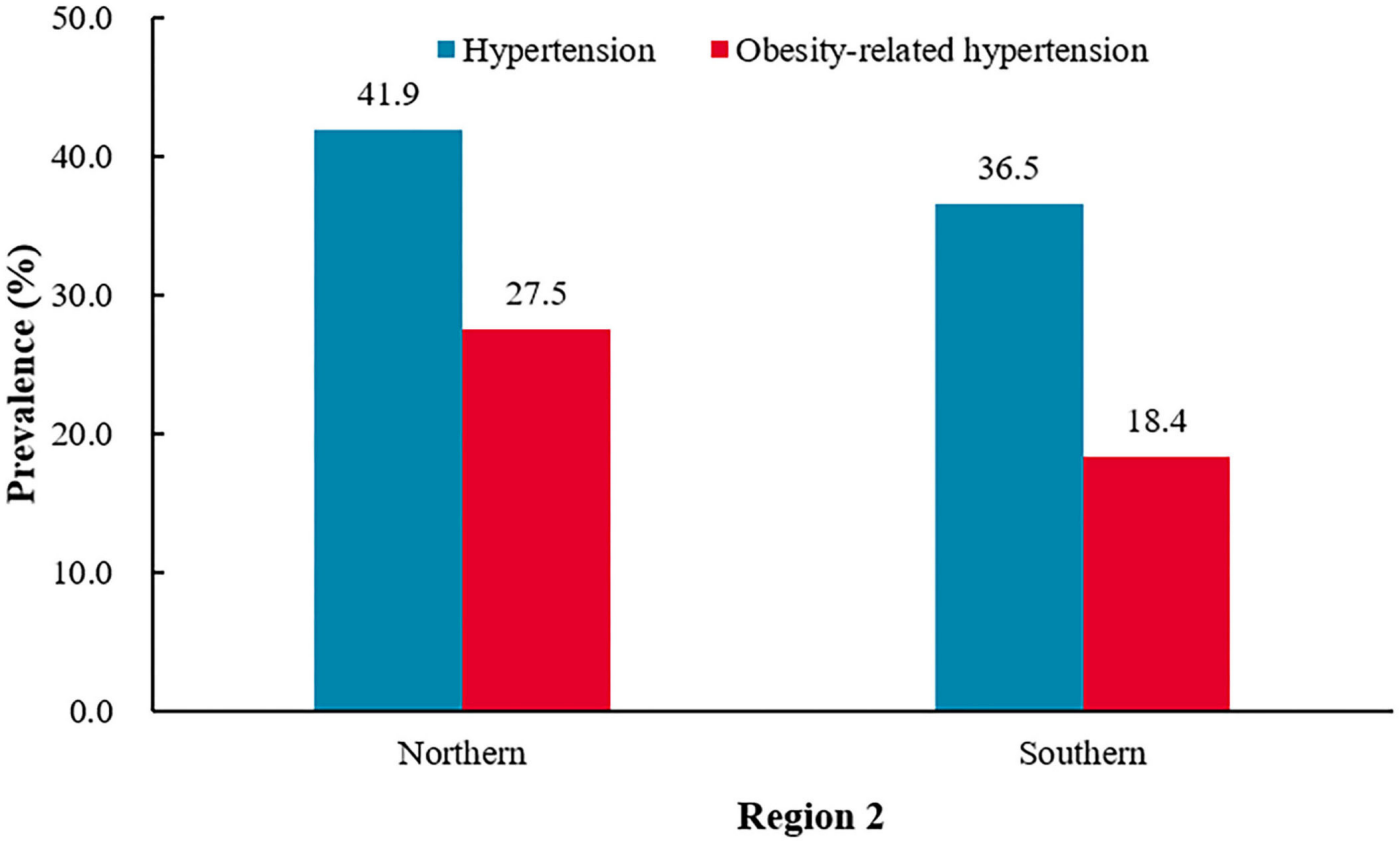
The prevalence of hypertension and obesity-related hypertension among Northern and Southern provinces.^†^*P* < 0.0001 for the difference in the prevalence of hypertension between Northern and Southern provinces.^‡^*P* < 0.0001 for the difference in the prevalence of hypertension obesity-related hypertension between Northern and Southern provinces.

Compared with the southern province residents, the northern province residents had higher prevalence of hypertension (northern 41.9% vs. southern 36.5%, *P* < 0.0001), obesity (northern 54.7% vs. southern 40.6%, *P* < 0.0001), and obesity-related hypertension (northern 27.5% vs. southern 18.4%, *P* < 0.0001). The prevalence of obesity-related hypertension was higher in northern provinces residents among the hypertensive participants (northern 65.7% vs. southern 50.2%, *P* < 0.0001), and it was also higher in northern provinces residents among the obesity participant (northern 50.3% vs. southern 45.3%, *P* = 0.0005) (Figure 5 and Table 4).

### Baseline characteristics of the non-obesity-related hypertensive and obesity-related hypertensive patients

The obesity-related hypertensive patients had a higher level of DBP, weight, WC, FBG, HbA1, TC, TG, LDL-C, and SUA and had lower level of age and HDL-C (all *P* < 0.0001, Table 5). The obesity-related hypertensive patients had a higher prevalence of diabetes mellitus, dyslipidemia, and hyperuricemia (all *P* < 0.0001, Table 6).

**Table 5:** Basic characteristics of the non-obesity-related hypertensive and obesity-related hypertensive patients.

**Variable**	**Overall** **(*N* = 3,951)**	**Non-obesity-related hypertension**				**Obesity-related hypertension**				***P*** **-value**
		**Overall** **(*N* = 1,652)**	**Men** **(*N* = 1,014)**	**Women** **(*N* = 638)**	***P-*** **value**	**Overall** **(*N* = 2,299)**	**Men** **(*N* = 883)**	**Women** **(*N* = 1,416)**	***P*** **-value**	
Age, years	63.2 ± 9.4	64.6 ± 9.5	64.8 ± 9.2	64.2 ± 10.1	0.1700	62.3 ± 9.2	61.7 ± 8.9	62.6 ± 9.3	0.0164	<0.0001
SBP, mmHg	145.3 ± 18.6	146.0 ± 19.1	146.2 ± 18.7	145.6 ± 19.8	0.5100	144.8 ± 18.2	144.5 ± 18.1	145.1 ± 18.2	0.4298	0.0622
DBP, mmHg	83.5 ± 11.9	82.8 ± 12.3	83.9 ± 12.5	81.1 ± 11.7	<0.0001	84.0 ± 11.6	86.1 ± 12.0	82.7 ± 11.1	<0.0001	0.0034
Height, cm	158.0 ± 8.6	158.1 ± 8.6	162.7 ± 6.6	151.0 ± 6.3	<0.0001	157.9 ± 8.6	165.5 ± 6.1	153.2 ± 6.1	<0.0001	0.4871
Weight, kg	62.0 ± 11.2	54.8 ± 8.6	58.0 ± 7.9	49.6 ± 7.0	<0.0001	67.2 ± 9.9	73.7 ± 7.7	63.2 ± 8.9	<0.0001	<0.0001
WC, cm	89.1 ± 10.2	79.8 ± 6.3	80.8 ± 6.6	78.2 ± 5.4	<0.0001	95.8 ± 6.6	97.4 ± 5.6	94.8 ± 7.0	<0.0001	<0.0001
FBG^*^, mg/dl	97.3 (88.3-106.3)	93.7 (86.5-102.7)	93.7 (86.5-102.7)	93.7 (86.5-102.7)	0.9525	99.1 (90.1-111.7)	99.1 (90.1-111.7)	99.1 (90.1-109.9)	0.4448	<0.0001
HbA1^*^, %	5.9 (5.6-6.3)	5.8 (5.5-6.1)	5.7 (5.5-6.1)	5.8 (5.5-6.1)	0.0015	5.9 (5.6-6.4)	5.9 (5.6-6.4)	6.0 (5.7-6.4)	0.0118	<0.0001
TC^*^, mg/dl	187.6 ± 37.4	184.0 ± 37.8	178.5 ± 36.4	192.9 ± 38.4	<0.0001	190.2 ± 36.8	182.7 ± 36.0	194.8 ± 36.6	<0.0001	<0.0001
HDL-C^*^, mg/dl	51.0 ± 11.9	54.7 ± 13.4	54.2 ± 14.0	55.4 ± 12.5	0.0639	48.3 ± 9.9	45.6 ± 9.3	50.0 ± 9.9	<0.0001	<0.0001
LDL-C^*^, mg/dl	104.3 ± 29.9	102.3 ± 30.5	99.1 ± 29.7	107.4 ± 31.2	<0.0001	105.8 ± 29.3	102.2 ± 28.5	108.1 ± 29.5	<0.0001	0.0003
TG^*^, mg/dl	123.0 (87.6-179.6)	98.2 (75.2-140.7)	93.8 (71.2-134.5)	107.1 (80.5-152.2)	<0.0001	144.2 (103.5-209.7)	140.7 (99.1-208.4)	145.1 (106.2-209.7)	0.1567	<0.0001
SUA^*^, mg/dl	5.2 ± 1.5	5.0 ± 1.5	5.5 ± 1.4	4.3 ± 1.2	<0.0001	5.3 ± 1.5	5.9 ± 1.5	4.8 ± 1.3	<0.0001	<0.0001

Values are presented as mean ± standard deviation, SD; TG, FBG, HbA1 were reported as median (interquartile range). WC, waist circumference; SBP, systolic blood pressure; DBP, diastolic blood pressure; FBG, fasting plasma glucose; TG, triglyceride; TC, total cholesterol; LDL-C, low density lipoprotein cholesterol; HDL-C, high density lipoprotein cholesterol; SUA, serum uric acid.

* Missing values.

**Table 6:** Comorbidities in the obesity-related hypertensive and non-obesity-related hypertensive patients.

**Variable**	**Overall** **(*N* = 3,951)**	**Non-obesity-related hypertension**				**Obesity-related hypertension**				***P*** **-value**
		**Overall** **(*N* = 1,652)**	**Men** **(*N* = 1,014)**	**Women** **(*N* = 638)**	***P-*** **value**	**Overall** **(*N* = 2,299)**	**Men** **(*N* = 883)**	**Women** **(*N* = 1,416)**	***P*** **-value**	
Diabetes Mellitus	979 (24.8)	271 (16.4)	163 (16.1)	108 (16.9)	0.6485	708 (30.8)	260 (29.4)	448 (31.6)	0.2679	<0.0001
Dyslipidemia	1,759 (44.5)	529 (32.0)	310 (30.6)	219 (34.3)	0.1113	1,230 (53.5)	506 (57.3)	724 (51.1)	0.0039	<0.0001
Hyperuricemia	607 (15.4)	170 (10.3)	124 (12.2)	46 (7.2)	0.0011	437 (19.0)	208 (23.6)	229 (16.2)	<0.0001	<0.0001

Values are presented as n (%).

### Multiple logistic regression

Multiple logistic regression analysis was used to determine the factors associated with obesity-related hypertension. The analysis showed that female gender, living in urban areas, diabetes mellitus, dyslipidemia, and hyperuricemia were positively correlated with obesity-related hypertension. Compared with the southern province residents, the northern province residents were positively correlated with obesity-related hypertension. Compared with the western province residents, the central and eastern provinces residents were positively correlated with obesity-related hypertension. Among hypertensive patients, the higher age levels were negatively correlated with obesity-related hypertension (Table 7).

**Table 7:** Multivariable-adjusted odds ratios for the obesity-related hypertension.

**Variables**	**Odds ratios (95%*CI*)**
Sex	
Men	
Women	2.323 (1.887–2.861)
Age, years	
45–54	
55–64	0.812 (0.665–0.992)
65–74	0.644 (0.525–0.790)
≥75	0.466 (0.359–0.603)
Residence	
Urban	1.574 (1.325–1.868)
Rural	
Region 1	
Western	
Central	1.255 (1.051–1.499)
Eastern	1.428 (1.193–1.708)
Region 2	
Northern	1.819 (1.571–2.106)
Southern	
Diabetes Mellitus	1.908 (1.602–2.273)
Dyslipidemia	1.995 (1.723–2.309)
Hyperuricemia	2.418 (1.951–2.997)

## Discussion

This is the first national epidemiological investigation about the prevalence of obesity-related hypertension among Chinese adults according to the definitions set by the 2016 Chinese expert consensus on obesity-related hypertension management. From the 2015 CHARLS, we found that the prevalence of hypertension and obesity-related hypertension were 39.1 and 22.7%, respectively, among adults aged 45 years or older in China. This cross-sectional study revealed that 58.2% of hypertensive patients had obesity-related hypertension. Nearly 2 in 5 adults aged 45 years or older had hypertension, and nearly 3 in 5 hypertensive patients had obesity-related hypertension.

The prevalence of hypertension was higher in men (men 40.3% vs. women 38.0%, *P* = 0.0179). This result is similar to previous studies (9, 27). However, women had a higher prevalence of obesity-related hypertension (men 18.8% vs. women 26.2%, *P* < 0.0001), which is similar to our previous observation among people in the southwest China (5). The prevalence of obesity-related hypertension was higher in women among the hypertensive participants (men 46.5% vs. women 68.9%, *P* < 0.0001), this could be explained by the fact that women had 1.6 times higher prevalence of obesity than men (men 36.4% vs. women 56.8%, *P* < 0.0001).

Compared to rural areas, previous studies have shown that urban areas had significantly higher prevalence of hypertension (9, 10), but the gap was gradually shrinking (28). In this study, the prevalence of hypertension was similar between urban residents and rural residents (urban 39.1% vs. rural 39.1%, *P* = 0.9802), this is similar to a nationwide survey conducted from October 2012 to December 2015 in China, from which there was no significant difference of hypertension prevalence between urban and rural areas (27). But the prevalence of obesity-related hypertension was higher among urban areas (urban 26.5% vs. rural 21.5%, *P* < 0.0001), this may be explained by urban residents had higher prevalence of obesity (urban 56.2% vs. rural 44.5%, *P* < 0.0001). People living in cites were more likely to have less physical activity, more sedentary activities, occupational stress, and other social pressures.

There was north-south gradient in both obesity and hypertension in previous studies (27, 29–31), as well as in this study. Compared with the southern province residents, the northern province residents had a higher prevalence of obesity-related hypertension. This phenomenon may be due to lack of fresh vegetables and fruits, longer winter, lower outdoor temperature, less physical activity, and genome changes related to the climatic adaption in northern provinces (32–36).

The prevalence of obesity and hypertension increasing from the west to the east had been shown in previous studies (28–31). Our study also showed the same trend. From western provinces to the central provinces to eastern provinces, there was an increasing trend in prevalence of hypertension, obesity, and obesity-related hypertension. China is a developing country with rapid economic development and urbanization, but the economic development has regional differences. From the west to the east, the state of economic development is gradually improving. Accompanying with economic development, the psychosocial stress and bad living habits were accumulated, which may cause hypertension and obesity (28, 37–40). Besides, both urbanization and migration from rural to urban areas are correlated with an increase in hypertension and obesity prevalence (41–46).

Age is a dominant risk factor for hypertension, as shown in previous studies (9, 47). In our previous study among adults aged 40–79 years in southwest China, the obesity-related hypertension increased with age (5). But in this study, it peaked at ages 65–74 years and the prevalence of obesity-related hypertension among hypertensive participants decreased with age. Compared with non-obesity-related hypertension, the obesity-related hypertensive patients had a higher prevalence of diabetes mellitus, dyslipidemia, and hyperuricemia (all *P* < 0.0001), and both hypertension and obesity were risk factors for atherosclerotic cardiovascular disease (ASCVD) and premature death (48, 49). This may explain why the prevalence of obesity-related hypertension among hypertensive participants was decreased with age. The prevalence of diabetes mellitus, dyslipidemia, and hyperuricemia among obesity-related hypertensive participants were nearly two times compared with non-obesity-related hypertensive participants (30.8 vs. 16.4%, 53.5 vs. 32%, and 19.0 vs. 10.3% all *P* < 0.0001) and all of which were leading causes of ASCVD among developing countries (50–52). This reminds us to strengthen the screening and control of DM, lipids, and uric acid in obesity-related hypertension. In contrast to the rapid economic development and living standards improvement, there is a lack of knowledge of healthy behaviors and management in people (27), which may lead to the increased hypertension, obesity, and obesity-related hypertension burden in China. Chinese government has carried out a series of reforms to improve this situation, such as universal access to basic medical insurance, promoting physical activities, medical staff supporting in the rural areas, and so on. Some effective actions, such as dietary salt reductions, improved public awareness programs, free blood pressure screening, integration of hypertension management into routine primary care practices, were recommended by previous studies (53–55).

This study has some limitations. First, our study encompassed 28 provinces, but not Tibet, Ningxia, Hainan, Hongkong, Macao, or Taiwan. Second, since this is a cross-sectional study, the results cannot be used to conclude cause-and-effect relationship between the risk factors and obesity-related hypertension. Third, we did not use the 24-h ambulatory blood pressure monitoring and did not exclude secondary hypertension, which are too costly in a national survey. Fourth, we defined the obesity-related hypertension on only two surrogate variables, BMI, and WC. There might be some hypertensive patients with normal BMI or WC, but high fat mass or visceral fat mass.

### Perspectives

The burden of obesity-related hypertension in China among adults aged 45 years or older was substantial, involving ~120 million people, and it was higher in women than in men. The prevalence of obesity-related hypertension among all participants showed a north-south and east-west descending gradient. The prevalence of hypertension was similar between urban residents and rural residents, but the prevalence of obesity-related hypertension was higher among urban areas than rural areas. The prevalence of obesity-related hypertension among all participants was increased with age (*P* < 0.001), and peaked at ages 65–74 years, whereas in hypertensive participants, it was decreased with age. Compared to non-obesity-related hypertensive patients, the obesity-related hypertensive patients had higher prevalence of dyslipidemia, diabetes mellitus, and hyperuricemia. Aggressive strategies should be carried out to prevent and manage obesity-related hypertension.

## Data availability statement

The raw data supporting the conclusions of this article will be made available by the authors, without undue reservation.

## Ethics statement

The protocol of the blood-based biomarker sample collection study was approved by the Ethical Review Committee (IRB) of Peking University (IRB 00001052-11014). Email: charls_info@pku.edu.cn. The patients/participants provided their written informed consent to participate in this study.

## Author contributions

YZ and X-BH conceived, designed the study, and drafted the manuscript. W-QZ designed the study, analyzed the data, and critically revised the manuscript. W-WT, J-XL, and R-HX advised on the interpretation of results. T-DW and X-BH critically revised the manuscript and were responsible for the research. All authors contributed to the article and approved the submitted version.

## Funding

This study was supported by the Natural Science foundation of China (grants 91746205).

## Conflict of interest

The authors declare that the research was conducted in the absence of any commercial or financial relationships that could be construed as a potential conflict of interest.

## Publisher's note

All claims expressed in this article are solely those of the authors and do not necessarily represent those of their affiliated organizations, or those of the publisher, the editors and the reviewers. Any product that may be evaluated in this article, or claim that may be made by its manufacturer, is not guaranteed or endorsed by the publisher.

## Abbreviations

BMI: body mass index
WC: waist circumference
CHNS: China Health and Nutrition Survey
CHARLS: China Health and Retirement Longitudinal Study
SBP: systolic blood pressure
DBP: diastolic blood pressure
HbA1c: glycated hemoglobin
TC: total cholesterol
LDL-C: low-density lipoprotein cholesterol
HDL-C: high-density lipoprotein cholesterol
TG: triglyceride
SUA: serum uric acid
SD: standard deviation
CI: confidence interval.
